# AGRIS: providing access to agricultural research data exploiting open data on the web

**DOI:** 10.12688/f1000research.6354.1

**Published:** 2015-05-08

**Authors:** Fabrizio Celli, Thembani Malapela, Karna Wegner, Imma Subirats, Elena Kokoliou, Johannes Keizer

**Affiliations:** 1 Food and Agriculture Organization of the United Nations, Viale delle Terme di Caracalla, Rome, 00153, Italy; 2 Agro-Know, Vrilissia, 152 36, Greece

**Keywords:** Semantic Web, Linked Open Data, AGRIS community, agriculture, agricultural data

## Abstract

AGRIS is the International System for Agricultural Science and Technology. It is supported by a large community of data providers, partners and users. AGRIS is a database that aggregates bibliographic data, and through this core data, related content across online information systems is retrieved by taking advantage of Semantic Web capabilities. AGRIS is a global public good and its vision is to be a responsive service to its user needs by facilitating contributions and feedback regarding the AGRIS core knowledgebase, AGRIS’s future and its continuous development. Periodic AGRIS e-consultations, partner meetings and user feedback are assimilated to the development of the AGRIS application and content coverage. This paper outlines the current AGRIS technical set-up, its network of partners, data providers and users as well as how AGRIS’s responsiveness to clients’ needs inspires the continuous technical development of the application. The paper concludes by providing a use case of how the AGRIS stakeholder input and the subsequent AGRIS e-consultation results influence the development of the AGRIS application, knowledgebase and service delivery.

## 1.0 Introduction

In the last decade Semantic Web technologies have introduced changes into the way structured data is published, shared and consumed on the Web. The Web has become a powerful bedrock where emerging online applications use it as an infrastructure to exchange, query and link semantically related data and information ^1^. In order to take advantage of the prowess of the emerging Web, many repositories have adopted linked data principles making the vision of a semantic Web of data a reality ^2^. Overtime, two important roles of linked open data (LOD) have emerged: consuming and publishing data, thereby facilitating innovation and wider knowledge creation and sharing ^3^. The principle of linked data has been extensively described in publications and books ^4, 5^. There are still challenges faced in browsing, analyzing, reusing and consuming linked data by the research community, Semantic Web community and policy makers. The major fallacy ^1^ of these emerging technologies is that they assume that connectivity to data repositories and entity resolution services are always online and available.

In the agricultural domain, the Agricultural Information Management Standards (AIMS) Team of the Food and Agriculture Organization of the United Nations (FAO) has taken advantage of the possibilities of LOD in making agricultural data, information and knowledge accessible. Often-cited examples include the publication of AGROVOC ( http://aims.fao.org/vest-registry/vocabularies/agrovoc-multilingual-agricultural-thesaurus) as a linked data set ^6^, and the AGRIS database and application ^7^. AGRIS (the International System for Agricultural Science and Technology) is an initiative that was set up in 1974 by the FAO to make agricultural research information discoverable and globally available. Since then AGRIS has been collecting from more than 150 data providers located in more than 65 countries. AGRIS collects and disseminates bibliographic information on scholarly and scientific publications in agriculture and related subjects.

AGRIS today is a ‘global public good’ ^8^, built and maintained by a big community of data providers, partners and users. This is based on two overarching principles. Firstly, that AGRIS grants complete core access to data where users are allowed to download and use the content subject to an acceptable use policy ( http://agris.fao.org/content/acceptable-use-policy). Secondly, users are invited to give ideas on the development of AGRIS and its vision through e-consultations, stakeholder meetings, user surveys and feedback. This paper will briefly overview the recent developments in AGRIS and outline the latest technical implementations. The objective of this paper is to show the responsiveness of AGRIS to the community (clients’) needs and review the steps leading to the technical development and future direction of AGRIS.

## 2.0 The AGRIS mashup

Since December 2013, AGRIS has exposed its database as LOD, defining uniform resource identifiers (URIs) for bibliographic publications and allowing anyone to reuse the database also through a SPARQL endpoint. After an initial period where LOD opportunities were tested in the OpenAGRIS system ^9^, the AGRIS team decided to adopt LOD standards into the deployed system. The goal was to take advantage of the latent knowledge available in the AGRIS data, in order to automatically discover and display related and relevant information from the Internet. AGRIS seeks to become the prime information service for agricultural research, where domain experts, agricultural extentionists, students, researchers, librarians/ information managers and decision makers can discover needed information with precision and recall it in a short response time. When the user is searching for a publication, the AGRIS system is able to enrich the user’s query by displaying a mashup page with results of related information available on the same topic. To achieve this, AGRIS adopted a dual approach that allows users to access agricultural information through:

- *Bibliographic metadata* in the domain of agricultural science and technology are stored in a central database, currently storing nearly 8 million bibliographic references of scholarly and scientific publications.
- *Other types of information* (distribution maps, passport data, pictures, other bibliography, etc.) that are interlinked to the AGRIS central database.

Two things are crucial to build a useful mashup page: the selection of the data sources and the precision of the automatic extracted resources. Precision in this context means the relevance of displayed resources ^10^ must be of the same subject coverage as that of the article selected by the user. In AGRIS this is possible through AGROVOC ^6^, which is a Simple Knowledge Organization System (SKOS) concept scheme used to index the AGRIS database. AGROVOC brings additional value as a thesaurus consisting of more than 32,000 concepts and is available in 21 languages, covering all areas of interest to the AGRIS database. Therefore, AGROVOC is the backbone of the resource discovery process where AGRIS records (which are indexed with AGROVOC concepts) are used to query external Web services (e.g. by scientific names) and SPARQL endpoints by using AGROVOC URIs or alignments with other thesauri related to agriculture. External data sources are identified based on the content, the relevancy to the AGRIS domain, and after evaluating, the information provider ^11^.

**Figure 1.**
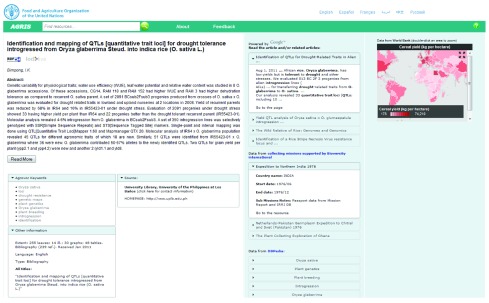
AGRIS mashup screenshot showing AGRIS bibliographic records pulling and visualising linked resources from external data sources.

Figure 1 shows a mashup page which displays an AGRIS record selected by the user with some AGROVOC descriptors and URIs (left). Once AGRIS loads the mashup page, it reads the list of AGROVOC URIs available within the AGRIS record, and run asynchronous queries to external Web services and SPARQL endpoints to get information related to the content of the selected AGRIS record. In the screenshot below for the AGROVOC concept “Oryza sativa”, AGRIS displays a distribution map from GBIF ( http://www.gbif.org; the Global Biodiversity Information Facility), as well as some germplasm collecting missions from Bioversity International ( http://www.bioversityinternational.org/). AGRIS pulls and visualizes data from World Bank, CGRIS germplasm database, and International Food Policy Research Institute (IFPRI). A full listing of external data sources AGRIS pulls from is available on AGRIS website ( http://agris.fao.org/content/how-it-works).

**Figure 2.**
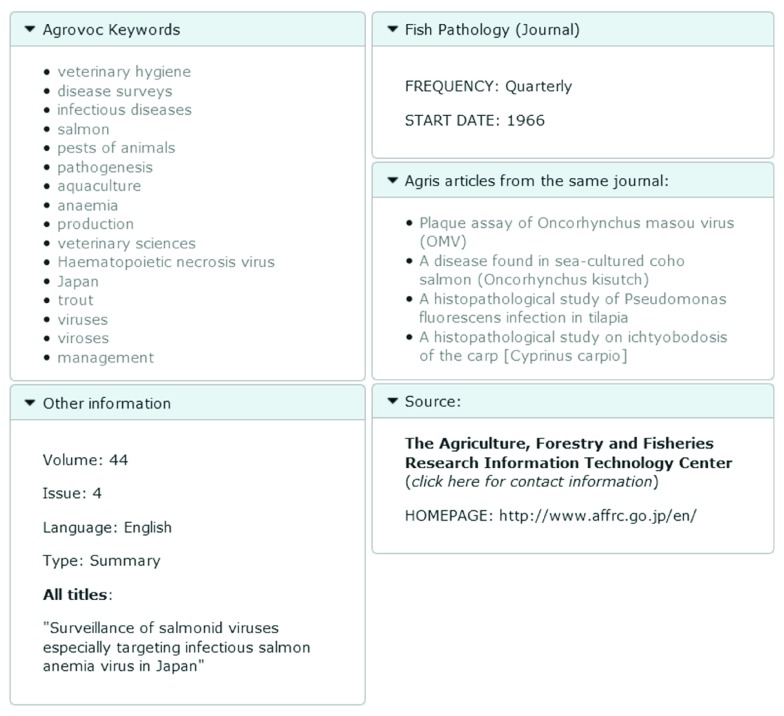
Additional bibliographic metadata available in the mashup page.

**Figure 3.**
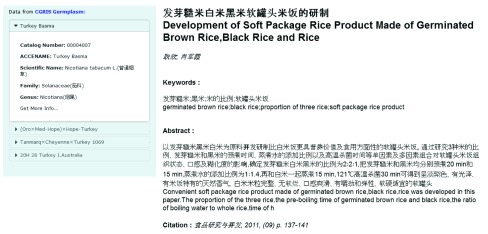
The CGRIS widget showing a record integrated from the Chinese Agricultural Scitech Documents Database. This allows users to tell the data source of the main bibliographic data of the AGRIS article.

## 3.0 AGRIS recent developments

AGRIS is constantly evolving to provide its users with new valuable services and many new different sources of information to be explored. The development of AGRIS, both on the service and data sides, is mainly driven by AGRIS users, who can provide feedback, ideas and needs in different ways: using the “ *feedback” form* available in the AGRIS Web site; responding to periodic surveys designed by the AGRIS team; sending emails to the AGRIS Team or joining online events like AIMS Webinars [ http://aims.fao.org/capacity-development/webinars] or AGRIS e-consultations. All this feedback is collected and analyzed to define priorities and provide new services to the community. For instance, after the adoption of a linked data infrastructure (when AGRIS and OpenAGRIS were merged at the beginning of 2014), the AGRIS team prepared an online survey and Webinars to collect feedback about the new AGRIS Web application. Two main activities were considered as top priorities to improve the service:

1. Inclusion of all the available bibliographic metadata in the AGRIS mashup page;
2. Multilingual search with the possibility to get results in several languages when searching with keywords in a specific language.

The first activity was carried out because, even though the AGRIS Web site mashes-up many sources of information to provide its users with a good browsing experience, for many AGRIS users access to the complete bibliographic metadata set is a valuable piece of information. Thus, the mashup view was extended to include all bibliographic metadata available, and advanced search functionality (namely, “classical view”) was re-introduced to allow filtering results according to specific metadata elements.

In the second activity, the multilingual search, the objective was to allow users to query the AGRIS database in their own native language, as well as retrieving results in different languages. This is exemplified by the following use case:

*Xian is a Chinese researcher and he wants to discover some knowledge from the AGRIS database. He wants to know something more about “rice” and recent research activities surrounding it, but he prefers to query the database using his own native language. So he starts querying AGRIS using the keyword “ *稻米*”. The AGRIS system discovers only 14 documents: they are not enough to add additional filters and they refer only to documents indexed with a Chinese keyword. Xian wants to access the international literature, so he also wants English articles. On the right side of the AGRIS interface, Xian enables the multilingual search and clicks on “GO”: 150,000 results! Maybe now Xian has too many articles to examine, but he can use other keywords to restrict the number of the results…*

The multilingual search is very important to facilitate access to literature in different languages: a future improvement of this feature will be offering the possibility of selecting sub-sets of languages to be included in the output of a query. The implementation of this feature relies on AGROVOC and on the AGRIS-linked open data infrastructure. In fact, while AGRIS records are indexed with AGROVOC keywords in a specific language, the translation to resource description framework (RDF) makes AGROVOC URIs usable. From an AGROVOC URI there is a possibility to extract labels of a concept in all the languages available in AGROVOC: those labels can be considered as “translations” of a query term, so that they can be used to expand the user’s query to include the translation of terms in different languages. To be more precise, the implementation of the multilingual search feature required two activities:

- Indexing AGROVOC URIs in Apache Solr ( http://lucene.apache.org/solr)
- Implementation of a software component that expands the user’s query to match results in all languages available in AGROVOC. The query expansion is transparent to the end user, who does not need to know technical details of this feature.

Another improvement was the inclusion of Chinese research content in the AGRIS database where a large amount of Chinese metadata were directly interlinked to the AGRIS database and displayed in the mashup pages. In the context of AgINFRA ( http://aginfra.eu/) and the collaboration between AGRIS and the Chinese Academy of Agricultural Sciences [ http://www.caas.cn/en/administration/research_institutes/research_institutes_beijing/77772.shtml], 500,000 resources from the Chinese Agricultural Sci-tech Documents Database (CASDD) and 410,000 resources from the CGRIS germplasm database were exposed as Web services and exploited as AGRIS external data sources, relying on the AGROVOC formal alignment with the Chinese Agricultural Thesaurus (CAT). The outcome of this activity was the inclusion of a large batch of Chinese research in agriculture in the AGRIS system, together with a unique collection of all types of plant genetic resources information from China, enriching the AGRIS knowledge base.

## 4.0 AGRIS data ingestion

**Figure 4.**
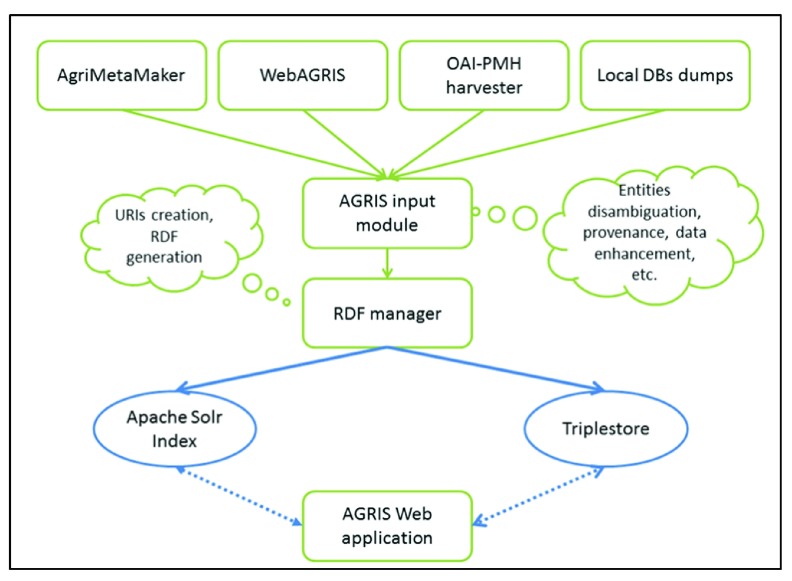
AGRIS dataflow and processing summarising different AGRIS data ingestion workflows and processing ^9^.

AGRIS is supported by a community of data providers, partners and users. AGRIS ingests bibliographic metadata provided by the community and publishes it as open data; the metadata is captured through either (i) pulling data through harvesting from clients or (ii) by data being pushed to the AGRIS from clients ^9^. AGRIS uses various tools and technologies to consume metadata from content providers and accepts any metadata records that meet the Meaning Bibliographic Metadata (M2B) standards. AGRIS’s data providers come from an international audience, with users often at varying stages of technological development. Figure 4 below summarizes the AGRIS data workflow, ingestion and processing.

The resultant AGRIS content is exposed via the AGRIS Web application – which is a mashup application that allows users to query the AGRIS content, interlinking all records to external sources of information. (See Figure 1 above and section 2.0 for more details).

## 5.0 AGRIS community needs

AGRIS’ vision is to be a responsive service to its global users’ needs by facilitating their contribution to the AGRIS core knowledgebase, AGRIS’s future and continuous development. Since 2014, FAO, Agro-Know ( http://www.agroknow.gr/agroknow/) and the Agricultural Information Institute of Chinese Academy of Agricultural Sciences ( http://www.caas.cn/en/administration/research_institutes/research_institutes_beijing/77772.shtml) (CAAS) have established a collaboration for the maintenance and centralization of AGRIS data processing. The collaboration is keen to keep AGRIS a community-driven product responding to the needs of the clients. The AGRIS team has a commitment to see AGRIS visitors and data providers as clients who contribute to the continuous development of AGRIS. In pursuit of this goal, periodic AGRIS stakeholder meetings, AGRIS e-consultations in the form of online surveys and user feedback are carried out to inform the development of the AGRIS application and coverage of the knowledgebase. Four **thematic areas** of focus have emerged since the initial discussions: 1.) AGRIS subject coverage, 2.) geographical accessibility of the system, 3.) improvement in user interactions and multilingualism and 4.) Strengthening the infrastructural backbone of AGRIS.

In considering these thematic areas, AGRIS partners agreed to map a strategy in each respective thematic area where the resultant output will drive technical developments, new functionalities and usability features. The involvement of the AGRIS community of users and further collaboration on technical developments will be invaluable in strengthening and developing new functionalities for the AGRIS portal. Feedback received from the community of data providers, partners and users is important for the possible improvements to the AGRIS portal and the knowledgebase. Furthermore, as stated earlier AGRIS has also been involved in a number of projects with the European Commission. For example, within the SemaGrow ( http://www.semagrow.eu/) project, AGRIS served as a demonstrator of a technical infrastructure based on the federation of many triple stores; relying on the two backend components Agro Tagger ( http://aims.fao.org/vest-registry/tools/agrotagger) and Web Crawler, AGRIS will be able to crawl the Web and to index discovered resources with AGROVOC URIs.

The AGRIS maintenance partners sought the engagement of the broader community into the further technical developments of AGRIS in the key thematic areas outlined above. The following issues emerged in the aforementioned four key areas:

### AGRIS content coverage

In terms of subject coverage, the AGRIS database collects bibliographic references in agriculture as defined by the FAO which includes nutrition, forestry, and fisheries. Since the nomenclature of AGRIS defines it as an international system for *Agricultural Science* and *Technology,* technology could also be included. The full list of subject categories can be downloaded online ( http://www.fao.org/scripts/agris/c-categ.htm): they will be revised in the coming months to enable the list to cope with the increased subject coverage requirement.

In terms of content, AGRIS core data initially focused on grey literature and later came to include papers, reports and other content types. The partners felt that AGRIS backbone data should continue to be bibliographic metadata, but felt that linked data technologies should be fully exploited to allow the inclusion of other relevant content types. To further develop the coverage of AGRIS content and to prevent stagnation, the AGRIS team aims to work out a new adequate subject scope for the AGRIS knowledgebase and discover new sources of information and data in collaboration with community partners. There are possibilities of linking AGRIS with science blogs and automatically updated feeds, and of further strengthening the relationship between AGRIS and AgriFeeds ( http://www.agrifeeds.org/) (for example, http://esciencenews.com and other feeds from scientific presses and universities).

Using data mining, the AGRIS database could be a way to access already existing information. In the AGRIS e-consultation users expressed their demand for more additional data like statistics, multimedia, price data, daily crops prices etc. The user survey additionally underlined the high demand for accessing full text resources. The AGRIS team has already responded with the implementation of the mashup page that allows linking to full text resources in the internet. The AGRIS Team is aware of the potential in identifying relevant content to interlink with AGRIS core data ( http://aims.fao.org/activity/blog/aginfra-promotes-integration-biodiversity-information-agris). The work on providing even more full-text links and resources will be continued, with the possibility of enriching AGRIS metadata with newly discovered full-text links and of setting up a link-checking mechanism to remove broken links. There will be a need for AGRIS’s authors’ disambiguation and the initial option could be to use unique author identifiers, for example AGRIS intends to use the AgriVIVO’s ( http://aims.fao.org/vest-registry/tools/agrivivo) scientific profiles. Another interesting activity will be the analysis of AGRIS full-text links to extract relevant information, such as a database of pictures indexed with AGROVOC, which will help enrich the content of a specific paper and to allow the re-use of pictures for personal reports or research activities, subject to copyright.

### Geographical accessibility of the system and multilingualism

Although AGRIS can be accessed from anywhere in the world, it has been noted that there is a lack of good performance in some regions – especially in China and East Asia. This might be due to the fact that the front-end Web application is hosted only in Rome. The AGRIS team is aware of this challenge and is trying to look for a solution to geographical accessibility by collaborating with community partners. In the meantime, the possibility of having two replicas of the database in two continents to minimize this challenge is being considered. This solution will require resources (such as a system administrator in each replica), a synchronization mechanism and a networking mechanism to geographically serve users seamlessly from different places in the world.

The user survey which registered 279 respondents who confirmed the lack of performance in some geographic regions. The overall feedback on performance was positive: around 80% of the users rated the performance of the web portal as extremely good or moderately good. Half of the users that are not satisfied with the AGRIS performance come from Asian countries. Several options to improve performance especially for countries in East Asia are currently being discussed and need testing. The AGRIS team must ensure that a better connection to Asia will not put other regions at a disadvantage. Recently, the multilingual search is an example of a client-demand service that has already been implemented. (see recent developments in section 3.0 above).

### Improvement in user interactions

The creation of a user registration facility is one of the main goals of the AGRIS team and was demanded by the community. Both AGRIS partners and users expressed their interest for a private area that allows the creation of personal profiles and the customization of the AGRIS interface (a step towards “social AGRIS”). The implementation of functions to define the portal design and select preferred datasets in the mashup page are possible as well as the addition of social functions like comments, ratings and quoting. The AGRIS team sees potential in having AGRIS sparking debates and collaborations based on AGRIS content. Support for mobile devices (e.g. smartphones and tablets) is another possible improvement that has been demanded in the surveys and discussed with individual community partners. In the survey users regard mobile device accessibility as very important with 40% of respondents wanting AGRIS to be read on a Tablet and 24% wanting access to AGRIS on their Smartphone.

### Strengthening the AGRIS backbone

The strengthening of the AGRIS backbone is important to provide a sustainable system. Currently, the AGRIS database is replicated in two types of models – (i) the AGRIS AP file system XML database and (ii) the AGRIS RDF triplestore. The AGRIS team will cease to maintain the AGRIS AP XML database and will design a new streamlined data model (most probably based on AGRIS RDF and linked open data-enabled bibliographic data (LODE-BD) ( http://aims.fao.org/lode/bd) that allows ingestion of data directly into the triple store. One of the goals is to design a more scalable and stable backend solution, as a system of load balancing of different instances of the AGRIS triplestore. In order to involve the community, the AGRIS team will have experiments as part of ‘Hackathons’ where participants can try different triplestore solutions simulating AGRIS queries to the database. 

The above summarized feedback represents the many suggestions of the clients’ needs and expectations from AGRIS. The value of the user-driven and responsive service is a core part of the AGRIS Vision and its continuous development. The move to a ‘social AGRIS’ will ensure that the AGRIS community of partners, data providers and users shape AGRIS service into the future. The collection of feedback from AGRIS spurs a number of potential enhancements now and in the future, with experiments made possible by the community (in the form of Hackathons). Redesign of workflows and AGRIS architecture, alongside strategic collaboration with global partners are some of the evident processes and activities within the AGRIS future vision.

## 6.0 Conclusion

AGRIS seeks to be a technological service that embraces the linked open data technologies while continuing to be a service relevant to its clients. This paper establishes that AGRIS is a global good, in that it is truly global in terms of data contribution and access, and also a public good ** in that it is built, maintained and responds to its community of partners, data providers, and users. AGRIS has been a bedrock for a number of semantic tools (as exhibited by the SemaGrow project) yet also provides a gateway to scientific research in Agriculture, Science and Technology. Semantic Web features have afforded AGRIS the ability to continue to be ‘the’ portal in Agriculture, Science and Technology for students, researchers and policy makers while at the same time constantly providing new and valuable services to the community in a dynamic and changing world.
