# Supplementary material for: AGRIS: providing access to agricultural research data exploiting open data on the web
Source: F1000Res. 2015 May 8;4:110. [Version 1] doi: 10.12688/f1000research.6354.1 (PMC4544375; doi:10.12688/f1000research.6354.1)
Supplement: Peer review history

# Peer review: Referee response for version 1

**DOI:** 10.5256/f1000research.6813.r9982

**Published:** 2015-08-18

**Authors:** Robert Davey

**Affiliations:** 1 The Genome Analysis Centre, Norwich, UK

I, Robert Davey (TGAC, UK)

- will sign my name to my review
- will review with integrity
- will treat the review as a discourse with you; in particular, I will provide constructive criticism
- will be an ambassador for the practice of open science

The authors describe AGRIS, a metadata repository for an impressive cohort of bibliographic linked open data in the agricultural sciences.

Managing large-scale data generative approaches ("big"/heterogeneous data) is only one side of the coin in the modern research era. With so much data available now (and this is only going to get worse), we need systems like AGRIS to make sense of the descriptions of data to ensure that researchers can find and reuse findings more easily, and more importantly integrate them into their own work.

Whilst I too found the AGROVOC system to be a little slow and unresponsive, the AGRIS portal is relatively fast which shows the underlying power of the linked datasets, which the mashup interface represents nicely. I did attempt to use the LOD Live portion of the site, but all of the resources I tried to visualise came up with the "no resource endpoint configured". I'm not sure if this is a factor of the actual resource itself, or an issue with the query mechanism. Could the authors give some working examples?

The paper mentions the use of Lucene, but are detailed technical documents about the software implementations that power the website available? Likewise, is there any relevant source code that would be suitable for release to the community? If so, links to this information might be useful.

The paper reads well, and gives some insight into how such a platform is built, assessed and used. The ability to search for terms in multiple languages is something that is incredibly important, and should be commended.

My only minor comments for revision would be that:

- the term "mashup" might not be well understood by many. A short description or refactoring of the term might add some clarity.
- I don't see the benefit of the 1.0, 2.0, etc section names. There are no subsections, so the article doesn't really need the ordered list style.

I have read this submission. I believe that I have an appropriate level of expertise to confirm that it is of an acceptable scientific standard.

# Peer review: Referee response for version 1

**DOI:** 10.5256/f1000research.6813.r8691

**Published:** 2015-05-18

**Authors:** Laura Privalle

**Affiliations:** 1 Regulatory Science Seeds and Traits Innovation Center, Bayer CropScience LP R&D, Morrisville, NC, USA

This is an informative article on the availability of a potentially very useful software tool of which I was not previously aware. The article presents clear examples of how to use the tool and the value it brings to the user. Others will be equally interested to learn of this website.

I have read this submission. I believe that I have an appropriate level of expertise to confirm that it is of an acceptable scientific standard.
